# Complement factors as biomarkers in ANCA-associated vasculitis in remission

**DOI:** 10.1093/cei/uxaf037

**Published:** 2025-05-29

**Authors:** Rebecca Trattner, Maria Iordanou, Sophie Ohlsson, Myriam Martin, Mårten Segelmark, Anna M Blom

**Affiliations:** Department of Translational Medicine, Division of Medical Protein Chemistry, Lund University, Sweden; Department of Clinical Chemistry and pharmacology, Office for Medical services, Region Skåne, Sweden; Department of Clinical Sciences, Division of Nephrology, Lund University, Skåne University Hospital, Sweden; Department of Clinical Sciences, Division of Nephrology, Lund University, Skåne University Hospital, Sweden; Department of Translational Medicine, Division of Medical Protein Chemistry, Lund University, Sweden; Department of Clinical Chemistry and pharmacology, Office for Medical services, Region Skåne, Sweden; Department of Clinical Sciences, Division of Nephrology, Lund University, Skåne University Hospital, Sweden; Department of Translational Medicine, Division of Medical Protein Chemistry, Lund University, Sweden; Department of Clinical Chemistry and pharmacology, Office for Medical services, Region Skåne, Sweden

**Keywords:** anti-neutrophil cytoplasmic antibody (ANCA)-associated vasculitis AAV, remission, complement, biomarkers

## Abstract

**Background:**

Anti-neutrophil cytoplasmic antibody (ANCA)-associated vasculitis (AAV) is a group of rare, autoimmune diseases causing inflammation in the vessel wall. Many organs can be affected, and kidney involvement is a common and serious manifestation. Complement activation is important in disease development and has also been detected in patients in remission. The reason for increased complement activation also without active disease is not understood.

**Methods:**

In this study, 65 AAV patients in remission, contributing with a total of 147 plasma samples, were included. Biomarkers of complement activation such as C4d (classical and lectin pathways), C3bBbP (alternative pathway), and soluble terminal complement complex (sTCC) (common terminal pathway) were measured with ELISA. For C4d measurement, an improved assay with an antibody targeting a cleavage neoepitope solely exposed during complement activation was used.

**Results:**

Our first hypothesis was that patients prone to recurrent flares might have an increased complement activation even when beeing in remission. However, we found no significant difference between those who did and did not develop flares during follow-up, nor any correlation between the total number of flares and any of the complement biomarkers. Interestingly, higher sTCC levels in remission correlated significantly to kidney involvement at the time of diagnosis and plasma creatinine levels at the time of sampling. Also, the diagnosis of microscopic polyangiitis (MPA), compared to granulomatosis with polyangiitis (GPA), yielded higher sTCC levels, and plasma C-reactive protein levels correlated significantly to sTCC.

**Conclusion:**

These findings suggest that persistent complement activation during remission in AAV may reflect underlying disease severity and organ involvement rather than predicting future flares.

## Teaser

This study investigated complement activation in 65 ANCA-associated vasculitis (AAV) patients in remission, measuring biomarkers such as C4d, C3bBbP, and sTCC. Results showed no correlation between complement activation and the likelihood or frequency of disease flares, contradicting the initial hypothesis. However, higher sTCC levels in remission were significantly associated with kidney involvement, plasma creatinine levels, and disease subtype (MPA vs. GPA), suggesting that complement activation reflects underlying disease severity and organ involvement.

## Graphical Abstract

**Graphical Abstract:**
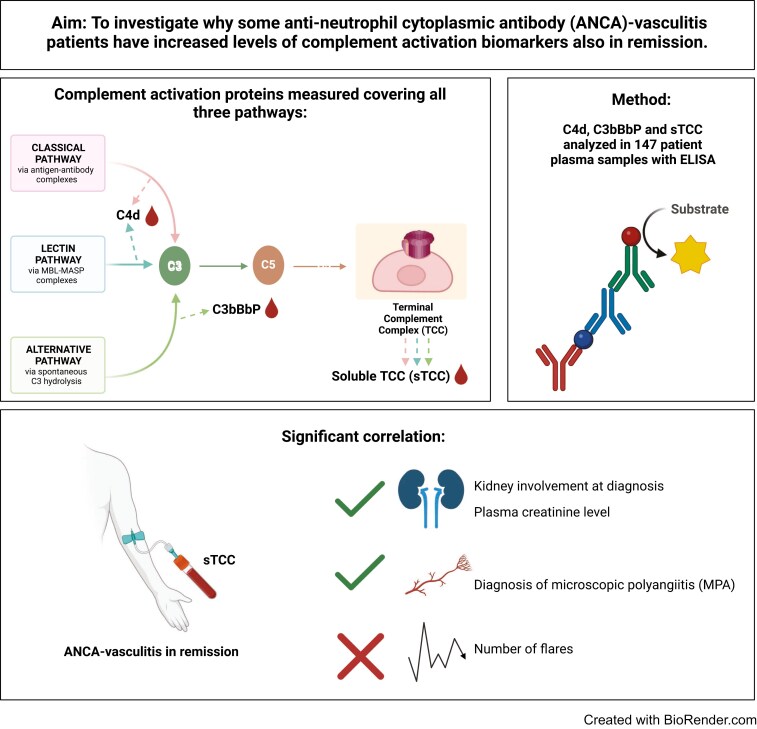

## Introduction

Anti-neutrophil cytoplasmic antibody (ANCA)-associated vasculitis (AAV) comprises a group of rare, often recurrent, and potentially life-threatening autoimmune diseases: granulomatosis with polyangiitis (GPA), microscopic polyangiitis (MPA) and eosinophilic granulomatosis with polyangiitis (EGPA). The main target autoantigens are proteinase-3 (PR3) and myeloperoxidase (MPO). Even though most organs can be affected, a majority of the patients have renal involvement and impaired kidney function is an important predictor of mortality in AAV [1–4].

Because of its pauci-immune appearance in kidney biopsies and absence of hypocomplementemia [5, 6], complement was for long not considered vital for AAV pathogenesis. This conception has over the years changed, with intensifying evidence of the pivotal role of this part of innate immunity in disease development from genetic analyses [7], animal models [8–10] and clinical *in vitro* [10] and *in vivo* patient studies [11–16]. In previous studies, a variety of complement proteins have been analyzed, with emphasis on alternative pathway activation. Genetic analyses of AAV patients presented an increased frequency of C4 allotype C4A3 and, in the subgroup of PR3-positive patients, of C3 allele C3F [7], suggesting complement involvement. In a mouse model using injection of anti-MPO IgG as an inducer of glomerulonephritis (GN), the development of crescentic GN was prevented in mice deficient of C5 (common complement pathway) and factor B (alternative complement pathway), while mice lacking C4 (classic and lectin complement pathways) developed disease to the same extent as wild-type mice [8]. Genetic analysis and plasma levels of complement factors in humans identified alternative pathway as a determining factor of disease susceptibility and prognosis [16]. These findings are in concordance with the growing body of evidence of complement involvement in AAV and indicate that classical and lectin pathways are not as essential as the alternative pathway.

Current treatment strategies for AAV involve immunosuppression, often including high doses of corticoids over prolonged periods of time, risking adverse effects commonly caused by this medication [3, 17]. There are currently six FDA-approved drugs targeting complement, and 40 additional drugs in Phase 3 clinical trials [18]. Complement inhibitor avacopan, a selective antagonist of the C5a receptor 1 (C5aR1, CD88), is already approved for use in combination with standard therapy. Avacopan has been suggested to replace high-dose corticoids in AAV with promising results [19–21]. Considering the increasing understanding of complement involvement in AAV, this group of drugs is of uttermost interest as a potential future treatment option, urging even more research in this area, not the least to evaluate biomarkers that could potentially be used to identify patients suitable for and to follow-up complement inhibiting therapy.

AAV is often recurrent, but patients may have long periods of remission without apparent disease activity. In some patients, flares never occur. There are no reliable tools used in the clinic to identify patients prone to additional flares. It is well known that patients suffering from AAV have increased complement activation during flares. Interestingly, some AAV patients have elevated plasma levels of complement activation products also without any signs of active disease [13–15]. In a cohort of MPO-ANCA positive AAV patients with renal involvement, samples were taken during active disease and in remission, and compared to age- and gender-matched healthy controls. Levels of C3a and C4d were significantly higher in remission compared to healthy controls, but only C3a levels were significantly increased in patients with disease activity in comparison to those in remission [13]. In another publication, plasma levels of Bb, C3a, and sC5b-9 in MPO-ANCA positive patients and C3a and C4d in PR3-ANCA positive patients in remission were significantly elevated compared to healthy controls, but there were notably no significant differences between levels of any complement activation biomarkers in active disease and remission [14]. When comparing the activation of complement in active disease and remission, levels of membrane attack complex (MAC) were significantly increased in active disease compared to the control group, but did not decrease when patients were in remission. In this cohort, only six of 24 patients had normalized MAC levels after achievement of remission [15]. The underlying cause driving or maintaining complement activation in remission is not known, and further investigation of this could increase the understanding of the pathogenesis of AAV.

To further assess complement activation in AAV in remission, we analyzed C4d, soluble terminal complement complex (sTCC), and C3bBbP in plasma samples and correlated the levels with clinical parameters and complementary laboratory tests routinely measured. This study aimed to investigate the reason why some patients have elevated levels of complement biomarkers even without active disease. Our main hypothesis was that increased activation of complement in remission might be related to an ongoing, low, disease activity, indicating an increased risk of flares. An alternative hypothesis was that specific organ involvement, for example renal impairment, might affect complement activation in different manners. Considering the importance of antibodies in the disease development of AAV, the classical pathway of complement activation would naturally be assumed to play a significant role, and is interesting to re-evaluate using an improved assay with an antibody against a cleavage neoepitope, only accessible during complement activation [22].

Addressing our first hypothesis, levels of complement activation products did not correlate to the number of flares in our cohort of AAV patients in remission. Further, investigating other causes of maintained complement activation in remission, demonstrated significantly increased plasma sTCC levels in patients who had kidney involvement compared to patients without kidney involvement at the time of diagnosis. At the time of sampling, levels of sTCC also correlated significantly with plasma creatinine and C-reactive protein (CRP) levels. Patients diagnosed with MPA had significantly higher sTCC levels in comparison to patients diagnosed with GPA. Hence, the increase of complement activation markers in AAV patients in remission might be due to the severity of the disease and involvement of specific organs but does not predict forthcoming flares.

## Materials and methods

### AAV patients and healthy controls

Outpatients at the Department of Nephrology at Skåne University Hospital in Lund, Sweden, were recruited from March 2015 to January 2019. Samples were taken during routine checkups with the aim of finding biomarkers predicting future flares of disease.

**Figure 1:**
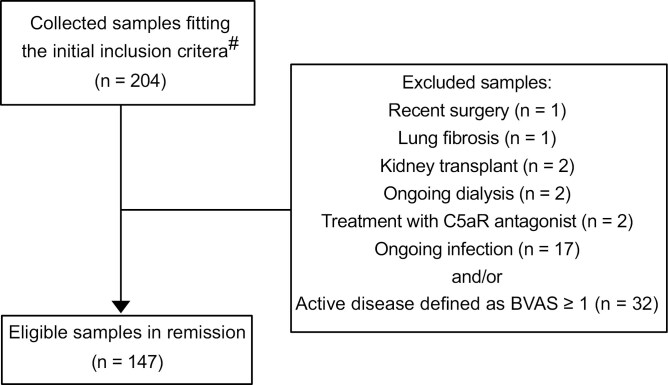
flow chart of sample exclusion. ^**#**^For each patient, a maximum of five samples was selected. The minimum time between two samples was three months

A total of 672 AAV samples with diagnosis of MPA and GPA were based on histopathological, radiological, laboratory, and clinical criteria, according to the EMA algorithm [23] were available. A maximum of five samples per patient was selected. The minimum time between two samples was three months, but preferably one year if possible. This selection resulted in 204 samples being analyzed. Clinical data were assessed retrospectively from patient records. Conditions expected to cause complement activation led to sample exclusion. Exclusion criteria were active disease defined as Birmingham Vasculitis Score (BVAS) version 3.0 [24] ≥ 1, dialysis, treatment with the C5aR-antagonist avacopan, ongoing infection, recent surgery, kidney transplantation or other diagnoses of serious disease, as indicated by Figure 1. BVAS score was assessed by the treating physician during the visit, and remission was defined as a BVAS score of 0. The exclusion criteria for ongoing infection were defined as symptoms of infection and increased level of CRP, decreasing after treatment with antibiotics, or infection verified with culture. Finally, 65 patients were included, contributing a total of 147 plasma samples (1-5 samples from each patient). Samples were aliquoted and rapidly frozen at −80°C, to avoid repeated freeze/thaw cycles. In Table 1, the demographics of AAV patients are shown. Plasma samples from 50 healthy controls were used to calculate the local normal reference range for the C4d and sTCC assays and from 28 healthy controls for the C3bBbP assay.

**Table 1:** demographics divided by AAV patients and samples

Characteristics	AAV patients *n* = 65	All samples *n* = 147
Samples per patient	2 (1-5)	
Age at sample collection		71 (22-92)
Time since diagnosis (years)		5.5 (0.2-40.2)
Females, *n* (%)	32 (49%)	
ANCA at diagnosis, *n* (%)		
Missing	0	
Negative	3 (5%)	
MPO	24 (37%)	
PR3	37 (57%)	
Both	1 (2%)	
ANCA at time of sampling, *n* (%)		
Missing		17 (12%)
Negative		46 (31%)
MPO		30 (20%)
PR3		54 (37%)
Diagnosis, *n* (%)		
MPA	26 (40%)	
GPA	39 (60%)	
Organ involvement at time of diagnosis, *n* (%)		
Kidney	52/63 (83%)	
Ear, nose, and throat	28/63 (44%)	
Pulmonary	28/63 (44%)	
Neurological	7/63 (11%)	
Skin	7/64 (11%)	
Eyes and oral cavity	5/63 (8%)	
BVAS at diagnosis	14 (5-22)^*a*^	
Creatinine (µmol/L)		105 (52-338)^b^
CRP (mg/L)		2 (0.5-48)^c^
Treatment		
No treatment, *n* (%)		29 (20%)
Only prednisolone, *n* (%)		11 (7%)
Other treatment^d^, *n* (%)		38 (26%)
Prednisolone + other treatment^d^, *n* (%)		69 (47%)
Prednisolone dosage (mg/day)		2.5 (0-25)

*Values are presented as median (range) unless otherwise stated.*

^*a*^ *3 missing;* ^*b*^ *5 missing;* ^*c*^ *8 missing;* ^*d*^ *Rituximab, Azathioprine, Mycophenolate mofetil, Methotrexate or Cyclophosphamide*.

Samples from patients and healthy controls were collected according to the Declaration of Helsinki and approved by the Regional Ethical Review Board in Lund (Dnr. 110/2008, Dnr. 2017/582). All participants provided written consent of participation, and patients were orally re-consented each time a repeat sample was drawn.

### Complement C4d and sTCC assessment with enzyme immunoassay

Levels of plasma C4d were measured using Complement C4d sandwich enzyme-linked immunosorbent assay (ELISA) (#COMPL C4d RUO, SVAR Life Science), and levels of plasma sTCC were measured using Complement TCC sandwich ELISA (#COMPL TCC RUO, SVAR Life Science). Plasma samples were pre-diluted 1:10 in PBS supplemented with 0.02% NaN_3_, 0.2% Tween 20, and 0.02 M Na_2_EDTA (AG buffer) in preparation for C4d assay and pre-diluted 1:3 in diluent included in the corresponding kit in preparation for sTCC assay. All samples were stored at -80°C until analysis, which was performed according to instructions from the manufacturer. Absorbances were read at 450 nm and 620 nm in Cytation-5 multi-mode reader (BioTek).

### Complement C3bBbP enzyme-linked immunosorbent assay (ELISA)

Levels of plasma C3bBbP were measured with sandwich ELISA, adjusted from the previously published protocol [25]. Plasma samples were pre-diluted 1:10 in AG buffer and stored at −80°C until analysis. A Maxisorp 96-well plate (Nunc) was coated with Murine Monoclonal Anti-Human Factor P #2 (A235, Quidel) and incubated overnight. As standard, ICS #2 (zymosan and heat aggregated IgG activated serum from Europe, 1000 CAU/ml) was used [26], diluted in AG buffer. The diluted ICS #2 was filtered and centrifuged using the Ultrafree-MC 0.22 µm GV Durapore Centrifugal Filter Units (UFC30GVOS, Merck Millipore Ltd) according to instructions (12 000 x g for 4 minutes). The standard curve started with a 1:25 dilution. AG buffer was used as blank and normal human plasma (pooled from six healthy donors) as a negative control. Patient EDTA plasma samples were further diluted to final dilution 1:25 in AG buffer before analysis. Standard, blank, negative control, and patient samples were added to the plate and incubated for 60 minutes at 4°C. Polyclonal Rabbit Anti-Human C3c (A0062, Dako) and then Polyclonal Swine Anti-Rabbit Ig/HRP (P0399, Dako) were added, each antibody incubated for 60 minutes at 37°C. Plates were washed with PBS supplemented with 0.2% Tween 20 in between steps. TMB one (4380A, Kementec) was used as a substrate to develop the plates and the reaction stopped after 25 minutes with 0.5 M H_2_SO_4_. Absorbances were measured at 450 nm and 620 nm in Cytation-5 multi-mode reader (BioTek). Normal human plasma was analyzed as a negative control in duplicates at each of the six ELISA plates. When using the mean normal human plasma C3bBbP level of each plate for the calculations, the inter-assay coefficient of variation is 71%.

### Standard laboratory tests

Plasma levels of creatinine and CRP were analyzed at the Department of Clinical Chemistry and Pharmacology, Office for Medical Services, Region Skåne, Sweden, according to routines at the time point of sample collection.

### Statistical analyses

Statistical analyses were performed in SPSS Statistics version 29 (IBM). Since 52 patients contributed with multiple samples, taken at different time points, calculations were carried out with repeated measures analysis using linear mixed models. The residuals of the complement biomarkers were not normally distributed, and therefore natural logarithmic values were used for all mixed model calculations. The P-values in the figures represent results from the repeated measures analysis using linear mixed models. To evaluate intraindividual variations of complement levels, the intraclass correlation coefficient was determined. In this statistical method, missing values are not allowed, meaning that all patients included need to have the same number of samples. To assess the consistency, absolute agreement with two-way random effects model, single measures was used. When evaluating the intraclass correlation, a coefficient between 0 and 1 is calculated, and an increasing number indicates higher agreement. Several values per patient give higher reliability, therefore intraclass correlation was calculated for the 22 patients contributing with at least three samples each. Correlation between the complement activation markers was calculated using Spearman’s rank correlation coefficient, including only the first sample from each patient. All figures were prepared in GraphPad Prism, displaying the actual complement activation levels to visualize the distribution more clearly.

## Results

### Complement activation during remission

**Figure 2:**
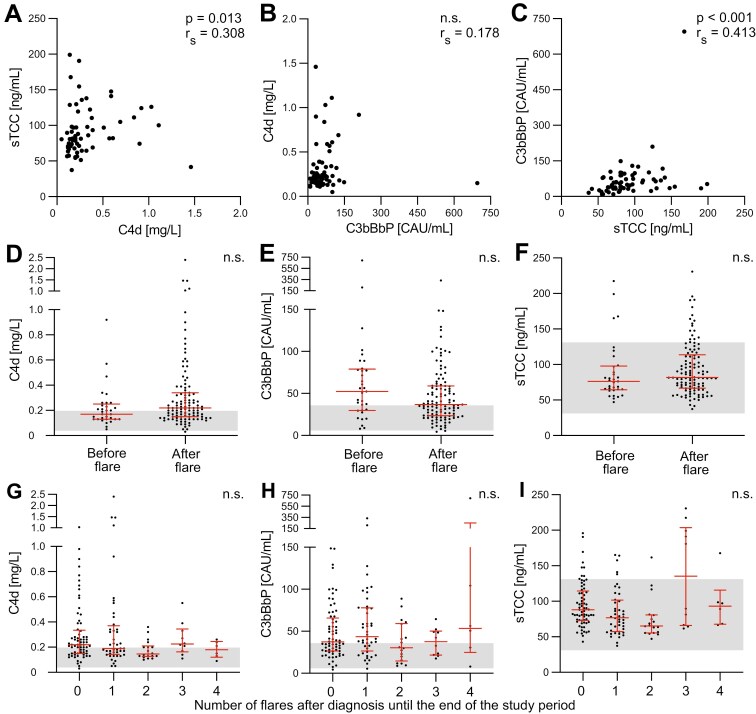
correlation among plasma C4d, C3bBbP, and sTCC levels and between biomarker levels and number of flares in AAV patients in remission. Correlation of complement activation markers including only the first sample from each patient (*n* = 65), calculated with Spearman’s rank correlation coefficient (A-C). Samples taken prior to flare from patients who did develop flares during the study period (*n* = 33) are compared with samples taken after flare (*n* = 114) (D-F). Correlation between complement activation markers and the total number of flares that had been recorded for the patient from diagnosis to the end of the study period (G-I). Graphs show a median with an interquartile range. Gray area is the local normal reference range for the complement analysis. Since some patients contributed with multiple samples, significance was calculated with repeated measures analysis using mixed models (D-I)

Including only the first sample from each patient, both levels of C4d and C3bBbP correlated significantly with sTCC (Spearman *r*_*S*_ = 0.308 and *P* = 0.013, and Spearman *r*_*S*_ = 0.413 and *P* < 0.001, respectively) (Figure 2A and C), indicating that different pathways are involved in the final generation of sTCC. There was no significant correlation between C4d and C3bBbP levels (Spearman *r*_*S*_ = 0.178) (Figure 2B). When measuring C4d levels, reflecting activity of the classical and lectin pathways, 83 (56%) of the 147 samples exhibited results above the reference range for healthy individuals. These 83 samples were obtained from 50 of the 65 patients. Assessing C3bBbP levels, also 83 (56%) of the 147 samples exhibited results above the reference range, and these samples were obtained from 51 of the 65 patients. Analyzing levels of sTCC, 21 (14%) of the 147 samples obtained from 15 of the 65 patients, exhibited results above the reference range.

### Complement activation in patients with and without subsequent flare

Of the 147 samples, 33 samples were taken prior to flare from the 16 patients that developed a flare during follow-up. The median time from sample to flare was 615 days (IQR 419-1183 days). This means that from the time point, at which the remaining 114 samples were drawn, no additional flare occurred during the follow-up. The median follow-up time from sampling was 1009 days (IQR 590-1346 days), and from diagnosis 3451 days (IQR 2278-5442 days). When comparing complement activation measured by plasma C4d, C3bBbP, and sTCC between these two groups, there were no significant differences for either of the biomarkers (Figure 2D-F). Henceforth, all samples from patients in remission were therefore combined regardless of sampling before or after diagnosis or flare.

### Complement activation in correlation to number of flares

To further address the hypothesis that increased complement levels in patients in remission are associated with flares, we examined the results based on the total number of flares recorded for each patient. There were no significant differences in complement levels between the number of flares for any of the biomarkers tested (Figure 2G-I). Table 2 summarizes the complement activation levels of AAV patients divided into groups depending on the number of flares.

**Table 2:** complement activation levels grouped by number of flares

Complement biomarker	C4d (mg/L)	C3bBbP (CAU/mL)	sTCC (ng/mL)
All samples *n* = 147 (65 patients)	0.21 (0.03-2.40)	38 (4-695)	81 (37-231)
No flares *n* = 69 (31 patients)	0.22 (0.03-1.03)	37 (4-149)	88 (43-196)
1 flare *n* = 44 (18 patients)	0.19 (0.05-2.40)	44 (5-333)	77 (37-165)
≥ 2 flares *n* = 34 (16 patients)	0.18 (0.09-0.55)	35 (8-695)	73 (47-231)
Mixed models*			
*P* value	0.215	0.241	0.522
No flares vs ≥ 2 flares, effect size (95% CI)	1.26 (0.89-1.79)	1.16 (0.86-1.56)	1.05 (0.86-1.28)
1 flare vs ≥ 2 flares, effect size (95% CI)	1.39 (0.95-2.04)	1.32 (0.95-1.82)	0.94 (0.75-1.17)

Levels of complement activation markers are presented as median (range) of the total number of samples. *Significance, effect size and confidence intervals were calculated with repeated measures analysis using linear mixed models. After statistical calculations, effect size and confidence intervals were transformed back from natural logarithmic values, giving a multiplicative factor.

### Intraclass correlation of complement levels in patients in remission

**Figure 3:**
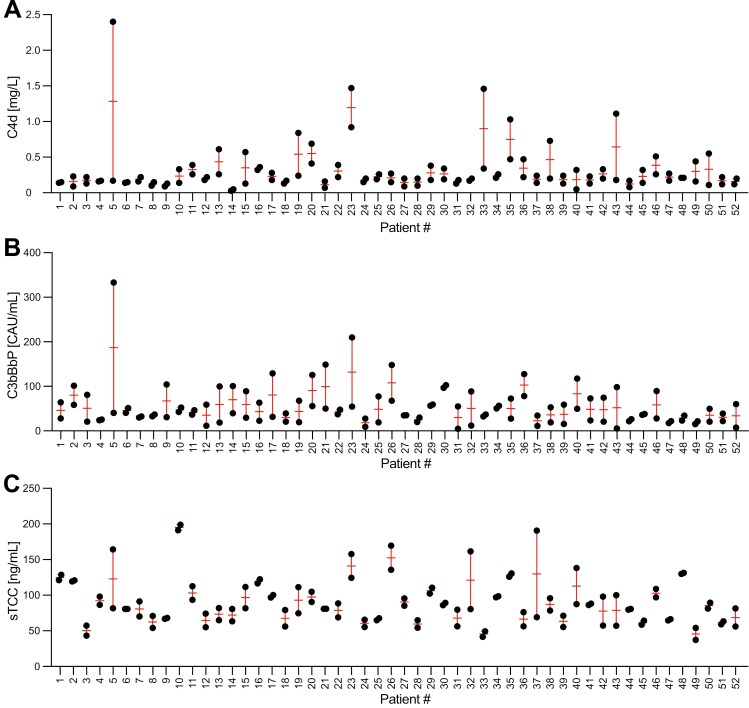
intraclass correlation of C4d, C3bBbP, and sTCC in AAV patients in remission. Plot of first and last samples of levels of C4d, C3bBbP, and sTCC in patients with multiple samples (*n* = 52) (A-C)

To assess the intraindividual variation of complement levels in AAV patients in remission, we calculated the intraclass correlation for the 22 patients who contributed with at least three samples. The intraclass correlation coefficient, evaluating the reliability, for sTCC was 0.66 indicative of a good congruence between the samples in an individual. For C4d, the intraclass correlation coefficient was 0.42, which could be interpreted as a fair concordance. C3bBbP did not show any conformity, with an intraclass correlation coefficient of 0.06. When plotting the first and last sample of all 52 patients with multiple samples, the levels of C4d (Figure 3A) and sTCC (Figure 3C) for each individual visually corresponded well between the two samples with some exceptions, but C3bBbP did not (Figure 3B).

### Correlation of complement activation levels to other clinical parameters

**Figure 4:**
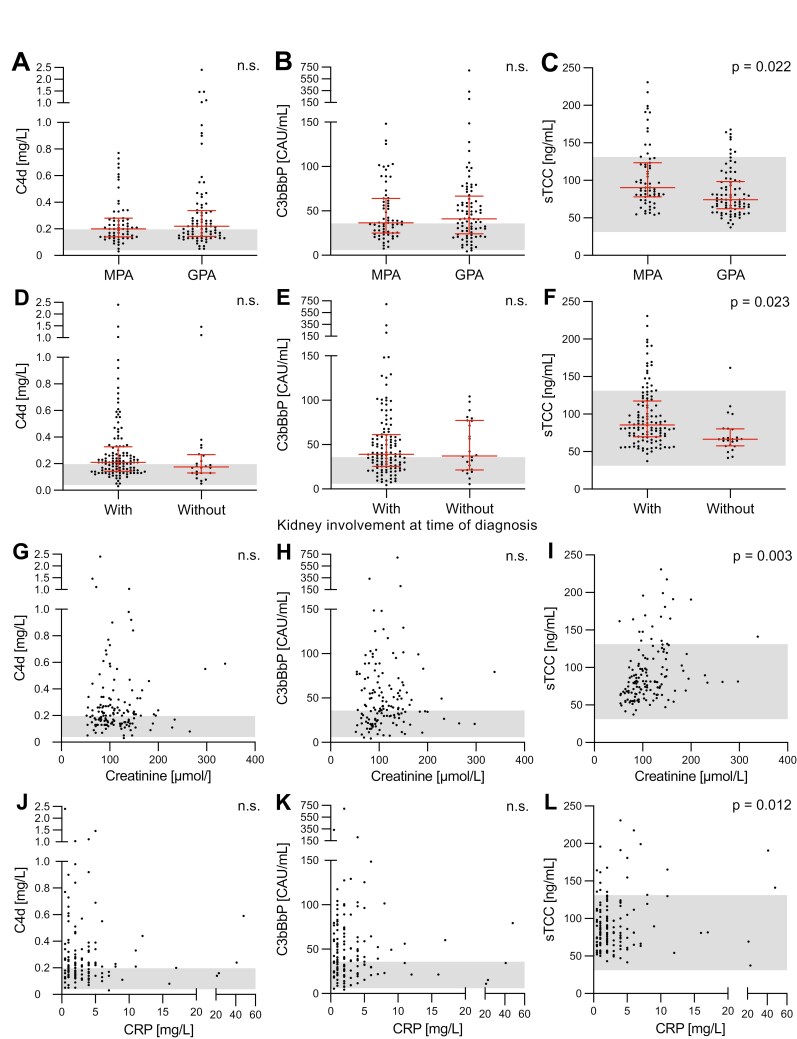
association of plasma levels of C4d, C3bBbP, and sTCC with clinical parameters and other biomarkers. Plasma C4d, C3bBbP, and sTCC levels in AAV patients in remission separated by diagnosis MPA or GPA (A-C) and kidney involvement at the time of diagnosis (D-F). Correlation of plasma levels of complement activation products with creatinine (G-I) and CRP (J-L). Graphs show the median with an interquartile range (A–F). The gray area is the local normal reference range for the complement analysis. Since some patients contributed with multiple samples, significance was calculated using repeated measures analysis using mixed models (A-L). MPA = microscopic polyangiitis, GPA = granulomatosis with polyangiitis

We detected significantly higher sTCC levels in plasma in patients with MPA compared to GPA (*P* = 0.022) (Figure 4C), whereas plasma C4d (Figure 4A) and C3bBbP (Figure 4B) did not differ significantly between the two diagnoses. Patients with kidney involvement at the time of diagnosis had significantly higher sTCC plasma levels in remission compared to patients without kidney involvement at diagnosis (*P* = 0.023) (Figure 4F). This pattern could not be observed either for plasma C4d (Figure 4D) or C3bBbP (Figure 4E). Levels of plasma creatinine and CRP were measured at the same time point as the sample collection for complement analyses. Interestingly, both creatinine and CRP had a significant positive correlation to sTCC plasma levels (*P* = 0.003 and 0.012, respectively) (Figure 4I and L). This association with creatinine and CRP could not be detected in C4d or C3bBbP plasma levels (Figure 4G, H, J, and K).

### Plasma complement levels in AAV patients in remission with respect to treatment

**Figure 5:**
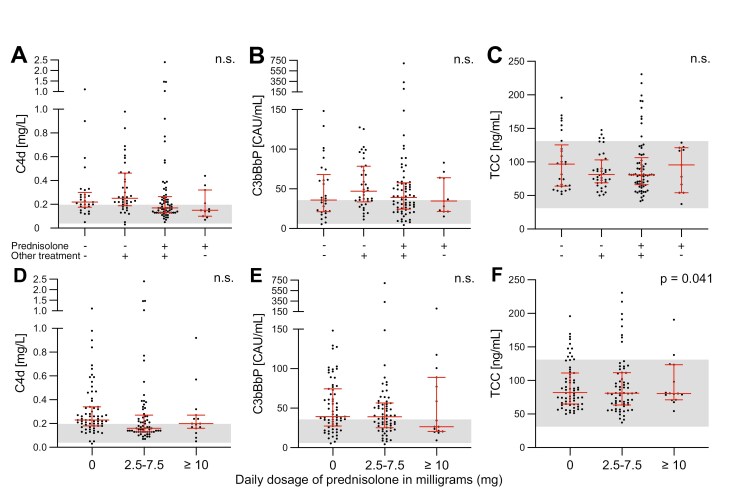
association of plasma C4d, C3bBbP, and sTCC levels with treatment in AAV patients in remission. Comparison of different treatment regimes (A-C) and of dosage of prednisolone (D–F). Graphs show the median with the interquartile range. The gray area is the local normal reference range for the complement analysis. Since some patients contributed with multiple samples, significance was calculated with repeated measures analysis using mixed models (A–F)

To investigate if there is a potential effect of treatment on plasma complement levels, patients with AAV in remission were divided into groups based on the category of administered drug (Figure 5A-C) and prednisolone dosage (Figure 5D-F) used during the time of sample collection. There was a tendency towards lower C4d plasma levels in patients treated with prednisolone, compared to patients without prednisolone treatment (Figure 5A and D), however, it was not statistically significant. The statistical calculations showed no significant difference, except for sTCC levels divided by prednisolone dosage (Figure 5F). The levels of sTCC were significantly increased in the group of patients with prednisolone treatment of ≥10 mg daily, compared to 2.5–7.5 mg or no prednisolone (*P *= 0.041). Notably, when removing the sample with the highest sTCC value in the group treated with ≥10 mg prednisolone daily (a patient with sTCC level 190.56 ng/mL) the result was no longer significant (*P *= 0.206).

## Discussion

This study confirms previous reports that patients with AAV have elevated levels of circulating complement activation products also during stable remission. Our primary hypothesis was that elevated complement levels might indicate ongoing subclinical disease activity with the potential of being used as biomarkers to predict future flares. However, we did not find any difference among samples taken from patients who during follow-up did develop flares and those who did not. Furthermore, there was no difference in levels between those with a history of multiple flares compared to those with no recorded relapse after treatment of the initial episode at the time of diagnosis. Due to the sample size and possible confounding effect of therapy, we cannot completely rule out a connection between subclinical disease activity and complement activation, but we can conclude that the assays used do not have future as biomarkers for predicting future flares.

Our alternative hypothesis was that complement activation during remission is linked to certain disease phenotypes or a specific organ manifestation. Our analyses of the intraindividual variation showed good or at least acceptable concordance for the C4d and sTCC assays, making analyses of correlations between disease manifestations at diagnosis and test results meaningful. A significant increase in plasma sTCC levels was detected in patients suffering from MPA, compared to GPA. When comparing MPO- and PR3-ANCA positivity at the time of sampling for complement analysis in patients in remission, there were no significances among the complement biomarkers. Since the samples were taken during remission, many patients have low or undetectable ANCA-titers, which might be an explanation. Also in active disease, a lack of association between complement biomarkers and ANCA has been reported [15], even though MPO is mainly associated with MPA and PR3 with GPA. It has been discussed whether ANCA positivity would be a preferable criterion to define homogenous groups of patients suffering from AAV, considering the impact of phenotype presented, response to therapy, and prognosis [27]. Although not directly comparable, considering the known correlation of ANCA positivity to diagnosis, our results are in line with a publication analyzing serum samples from a biobank, revealing higher complement levels in MPO-positive individuals compared to MPO-negative and PR3-positive prior to symptom onset [28]. One reason for the increased complement activation in patients suffering from MPA compared to GPA might be the difference in organ involvement. Different manifestations of disease might activate complement differently. Necrotizing GN is a common manifestation of MPA but also occurs in GPA [29–31], and kidney biopsies have been positive for immunoglobulins and complement [12, 32] even though it previously was considered pauci-immune. In our cohort, 25 of 26 patients with MPA had kidney involvement at the time of diagnosis. Of the 39 patients with GPA, only 27 had kidney involvement at the time of diagnosis (no data regarding organ involvement at the time of diagnosis in two GPA patients initially diagnosed in other regions of Sweden). Possible causes for increased complement biomarkers in patients in remission, that had renal manifestations at diagnosis, might be complement activation in the injured kidneys, or that the kidney impairment leads to general endothelial damage involving all vessels. The kidneys have several specific characteristics making them particularly susceptible to damage caused by complement activation. For example, the abundant renal blood flow leads to exposure of inflammatory mediators and activated agents generated elsewhere in the body, and the filtration in the glomerular capillaries increases the concentration of proteins. Complement factors may also be synthesized locally by the kidney [33], and the renal regulation of complement activity by inhibitors might be relevant. Further, fibrotic renal tissue may contribute to ongoing complement activity [34]. Also, though not yet identified, other subgroups of AAV could theoretically exist with specific complement activation patterns. Hence, serotype, phenotype, or specific organ involvement might all affect complement activation.

C4d is a biomarker of complement activation by the classical and lectin pathways, and by an assay developed by our group, it is easily measured in a stable way [22]. In comparison to C3 and C4, where levels represent the net rate of production and consumption, this assay measures a cleavage neoepitope solely exposed during complement activation and it has proven valuable as a biomarker in another autoimmune disease [35, 36]. Alternative pathway activation can be assessed by analyzing C3bBbP levels measuring the alternative pathway C3 convertase. By determining the level of the final step of general complement activation, sTCC, all three pathways are covered in our study. Many previous publications elucidating the role of complement in AAV used mouse models. To keep in mind, there is no animal model to study PR3-ANCA positive AAV [37] and also, the importance of different mechanisms to activate complement varies among species. In mice, the classical pathway may not be as central as it is in man, making the findings in mouse models complex to translate to human conditions. Levels of both C4d and C3bBbP correlated significantly with sTCC in AAV patients in remission, indicating that different pathways are involved in the final sTCC generation.

In SLE, we have already demonstrated that plasma C4d levels can be used to discriminate kidney involvement in terms of lupus nephritis and correlate to C4d kidney deposits [35], making kidney involvement also in AAV interesting to further investigate. In this cohort of AAV patients in remission, the significant increase of plasma sTCC levels in patients with kidney involvement at the time of diagnosis and significant correlation to current creatinine levels at the time of plasma sampling, indicating renal impairment, were interesting. Others have reported increased C5 levels compared to controls in AAV patients with renal involvement at the time of diagnosis [28]. Patients with renal involvement at diagnosis also have worse prognosis, compared to those without renal involvement [38]. When measuring complement levels in urine, levels of Bb correlated significantly with serum creatinine [12]. In a cohort of active AAV, there were no correlations between plasma complement levels tested and creatinine [15]. Complement involvement in patients with renal manifestations is not surprising, considering it is also seen in other autoimmune diseases e.g. IgA vasculitis and SLE [31, 35, 36]. CRP is an acute-phase protein synthesized by the liver, increasing during inflammation, often used to assess inflammation and infection. Also, CRP correlated significantly to sTCC in our cohort with patients in remission, in contrast to patients with active disease with no correlation between plasma complement levels and CRP previously reported [15]. Complement proteins and CRP are both elevated during inflammation, which logically explains their correlation.

Due to the sample size, we have limited power to detect differences based on treatments, as there are many different combinations of drugs. Visually, there is a tendency towards lower C4d levels in patients with prednisolone treatment, but this was not statistically significant. Because of the limited number of samples, we have not tested other potential cofactors such as age, sex, disease duration, other co-morbidities, smoking, and lifestyle. As far as we know, this is the first publication of an AAV study using the C4d assay from SVAR. This assay has proven more stable than prior ones, even with repeated freeze-thaw cycles of the samples, and uses an antibody against a neoepitope only accessible after complement activation [22]. Even if there were no significant correlations with the C4d biomarker in this cohort of patients in remission, it could be interesting to use this assay in future studies. It is well known that preanalytical errors are common during sampling, and by using the C4d assay from SVAR, less prone to artefacts, one could possibly minimize those errors and get more reliable results. When analyzing plasma samples from AAV patients with active disease (BVAS ≥ 1), we detected a significant correlation between C4d and BVAS (unpublished data). In previous studies, significant correlation between plasma Bb and BVAS [13] and, on the contrary, no correlations between the complement biomarkers analyzed and BVAS [14, 15] have been reported. It is worth noting that a different C4d assay was used in both of these publications.

Preanalytical handling of samples is of uttermost importance, especially for the analysis of C3bBbP requiring preferably fresh plasma samples not previously frozen. This might be the reason for the high inter-assay coefficient of variation for the C3bBbP assay. Recommendations of not freezing samples are practically challenging in research, but also in clinical practice at a hospital laboratory. Our samples were rapidly frozen at −80°C and aliquoted to minimize the freeze and thaw cycles but had to be frozen and thawed a few times for practicability. This could potentially have affected our insight into alternative pathway involvement specifically.

Since we have a varying number of samples from different patients, taken at uneven time points, repeated measures mixed models was used for all statistical calculations. Individual values can therefore be weighted differently, depending on the number of samples from one patient and also the specific place in the sampling sequence. This is illustrated by the statistical significance in Figure 5F, indicating higher plasma sTCC levels in patients with ≥ 10 mg prednisolone daily, compared to 2.5–7.5 mg or no prednisolone. This could be a random finding, due to multiple testing.

The diversity of complement activation markers, analyzed in different settings, sometimes generating contradictory results in the field of AAV research calls for a joint effort to investigate which factors could act as clinically relevant biomarkers. Keeping in mind that the complement inhibitor avacopan, targeting complement at C5a level resulting from all three activation pathways, is already used in AAV, response to treatment would be interesting to evaluate in relation to complement activation marker regardless of route of activation such as sTCC. Entering an era of personalized medicine, the use of biomarkers to optimize individualized treatment strategies is of great interest. Complement activation biomarkers could potentially be used to identify patients suitable for this expensive treatment, and dosage might be optimized to reach favorable complement suppression with minimized risk of adverse effects.

## Conclusion

It is a known phenomenon that a certain proportion of AAV patients in remission exhibit increased levels of complement activation markers, which was also the case in our cohort. We detected a significant increase in plasma sTCC in patients with kidney involvement at the time of diagnosis, and a significant correlation between sTCC and creatinine in plasma, in samples from patients in remission. Additionally, levels of sTCC were higher in patients with MPA, compared to GPA, and correlated significantly to plasma CRP levels. There were no correlations between levels of complement biomarkers C4d, C3bBbP, or sTCC in plasma and number of flares in AAV patients in remission. Thus, we think that the increased levels of complement activation products in some patients in remission are not caused by subclinical vasculitic disease activity, ergo are not biomarkers to use as indication of treatment adjustments to prevent forthcoming flares. However, the increased levels in remission could reflect ongoing renal interstitial scarring that might be responsive to pharmacological intervention.

## Data Availability

All results from the experiments and analyses of data are available upon request.
